# Ethics of primate use

**DOI:** 10.5194/asr-5-11-2010

**Published:** 2010-11-12

**Authors:** M. J. Prescott

**Affiliations:** National Centre for the Replacement, Refinement and Reduction of Animals in Research (NC3Rs), 20 Park Crescent, London, W1B 1AL, UK

## Abstract

This article provides an overview of the ethical issues raised by the use of non-human primates (NHPs) in research involving scientific procedures which may cause pain, suffering, distress or lasting harm. It is not an exhaustive review of the literature and views on this subject, and it does not present any conclusions about the moral acceptability or otherwise of NHP research. Rather the aim has been to identify the ethical issues involved and to provide guidance on how these might be addressed, in particular by carefully examining the scientific rationale for NHP use, implementing fully the 3Rs principle of Russell and Burch (1959) and applying a robust “harm-benefit assessment” to research proposals involving NHPs.

## 1 Introduction

### 1.1 NHP use in scientific procedures

NHPs are among the most extensively studied of all animals in the fields of behaviour, psychology, ecology, conservation and anthropology (see the PrimateLit bibliographic database: http://primatelit.library.wisc.edu/). They are also used in biomedical and biological research involving regulated scientific procedures, mainly in the fields of microbiology, immunology, neuroscience, biochemistry, pharmacology and toxicology, because their physiological and psychological similarities to humans make them high fidelity models^1^ (Carlsson et al., 2004; Hau et al., 2000; Weatherall et al., 2006). The majority are Old World monkeys (macaques, vervet monkeys and baboons), both purpose-bred and wild-caught; New World monkeys, Prosimians and Great Apes (chimpanzees, *Pan troglodytes)* are also used^2^ (Carlsson et al., 2004; Conlee et al., 2004; European Commission, 2007; Hagelin, 2004; Rennie and Buchanan-Smith, 2005). An estimated 100 000–200 000 NHPs are used annually world-wide, mostly in the United States of America (USA), the European Union (EU) and Japan (Carlsson et al., 2004). Currently their use within the pharmaceutical industry is rising in line with the increasing number of biopharmaceuticals entering the drugs pipeline (Chapman et al., 2010; Hobson, 2000).

### 1.2 Views on NHP use

The use of NHPs in scientific procedures is one of the most contentious issues in science. At the time of writing, this issue is high on the political agenda in the EU, following a proposal from the European Commission (2008) to revise *“Directive 86/609/EEC on the Protection of Animals Used for Experimental and Other Scientific Purposes”* (European Community, 1986) and an opinion from its Scientific Committee on Health and Environmental Risks (2009) on *“The need for non-human primates in biomedical research, production and testing of products and devices”*. The revision provides the opportunity for the EU Parliament and Council of Ministers to introduce new restrictions on NHP use. Although NHPs account for a very small proportion of the total number of vertebrate animals used in scientific procedures (0.09% of those used in the EU in 2005: European Commission, 2007), opinion polls repeatedly show a high level of concern about their use amongst the general public (European Commission, 2006; New Scientist/MORI, 1999; Pifer et al., 1994). For example, more than 80% of respondents to the Commission’s 2006 public consultation on animal experiments considered the use of NHPs to be not acceptable.

The consensus within the scientific community is that the close **phylogenetic** relationship of NHPs with humans makes them the best available animal models for particular research questions, and that, in the absence of suitable alternatives, their appropriate use remains essential in certain areas of biomedical and biological research and for the safety assessment of pharmaceuticals (Hau et al., 2000; Hobson, 2000; National Research Council, 2003; Scientific Committee on Health and Environmental Risks, 2009; Weatherall et al., 2006). Many antivivisection and animal welfare organisations, however, argue that it is this same relationship that causes them to object to NHP research, since it implies that NHPs can suffer in similar ways to humans. Furthermore, NHPs cannot consent to their own participation in research and, generally, will not benefit from such participation. For these reasons, the animal protection community believes that NHP experiments are unethical and should be banned or rapidly phased-out (Balls, 2000; Eurogroup for Animal Welfare, 2005; Humane Society of the United States, 2009; Thew and Seymour, 2009). Both communities have at times used the scientific literature selectively to support sweeping statements about the scientific validity, utility and moral acceptability of NHP use as a whole; this is irresponsible and misleading, and perpetuates entrenched and polarised viewpoints.

## 2 Ethical frameworks and legal controls applied to NHP use

The fundamental ethical dilemma raised by the use of NHP in experiments is the same as for the use of other animals: are we, human beings, morally justified in causing animals pain, suffering, distress and/or lasting harm in research aimed at alleviating or preventing human suffering, or furthering scientific knowledge. There is a wide spectrum of views on this issue in society, from those who consider all animal experiments to be immoral to those who believe few animal experiments to be unjustified if they benefit humans in some way (Nuffield Council on Bioethics, 2005). Moreover, many people hold varying opinions depending on the precise circumstances in question (e.g. the purpose of the research and the anticipated benefit, the species to be used, the level of harm caused to the animals involved). Therefore, most committees responsible for scrutinising the ethics of animal research proposals, whether at a institutional, local, regional or national level, aim to reach a collective decision, involving a diversity of perspectives and expertise (e.g. in the scientific area in question, animal welfare and ethics) (Animal Procedures Committee, 2009; de Greeve and de Leeuw, 1999; Home Office, 1998; Kolar, 2004).

### 2.1 Utilitarianism and the harm-benefit assessment

The approach to the ethical dilemma most often adopted is a pragmatic, utilitarian one. **Utilitarianism** requires us to strike the most favourable balance of benefits and costs for all the sentient individuals affected by what is proposed to be done. The underlying notion is that we can work out what is the ethical course of action by trading off one against the other (although this precept has been attacked by some moral philosophers). In the case of NHP research, the human interest in obtaining some benefit for mankind must be balanced against the interests of the NHPs in avoiding harm (Quigley, 2007).

This approach, referred to as a “cost-benefit assessment” or “harm-benefit assessment”, forms the cornerstone of the United Kingdom (UK) Animals (Scientific Procedures) Act 1986 (ASPA) (UK Government, 1986). The Act is unique in explicitly requiring a harm-benefit assessment of every application to the Home Office for a project licence to conduct animal research. The European Commission (2008) intends that a harm-benefit assessment be part of the ethical evaluation of research projects by national regulatory authorities under the revised Directive 86/609/EEC, and there have been calls for such an assessment to be applied to NHP use in the USA (Conlee et al., 2004)^3^.

### 2.2 The 3Rs

The moral acceptability of animal research is less questionable where animal use and suffering are minimised, in line with the 3Rs principle of Russell and Burch (1959):

- Replacement of animals with non-animal methods;
- Reduction of the number of animals used to obtain information of a given amount and precision;
- Refinement of scientific procedures and husbandry to minimise suffering and improve animal welfare.

In addition to its value as an ethical framework for humane experiments, the 3Rs principle has considerable scientific merit and receives tacit support from the general public in opinion polls on animal experimentation (Ipsos MORI, 2009). Hence, it features in most codes of conduct on animal research and, in developed countries at least, scientists are required by law to apply something like it (European Community, 1986; Shoji, 2008; UK Government, 1986; United States Department of Agriculture, 1990). For example, Article 7 of Directive 86/609/EEC states “*an experiment shall not be performed if another scientifically satisfactory method of obtaining the result sought, not entailing the use of an animal, is reasonably and practicably available”* and *“When an experiment has to be performed, the choice of species shall be carefully considered and, where necessary, explained to the authority. In a choice between experiments, those which use the minimum number of animals, involve animals with the lowest degree of **neurophysiological** sensitivity, cause the least pain, suffering, distress or lasting harm and which are most likely to provide satisfactory results shall be selected”*.

### 2.3 Responsibilities

There is broad support within the scientific and animal welfare communities for application of the 3Rs and a harmbenefit assessment to NHP research such that NHPs are only used in experiments where absolutely necessary (i.e. where no alternative methods are available), where morally justified, and where the numbers used and animal suffering are kept to the minimum (Boyd Group, 2002; Joint Working Group on Refinement, 2009; Scientific Committee on Health and Environmental Risks, 2009; Weatherall et al., 2006). Such judgements can only be made case-by-case for individual scientific objectives and projects (see Sect. 4) and usually involve the researcher, relevant ethics committee and/or national regulatory authority. Increasingly, public bodies funding NHP research, such as the European Commission, Medical Research Council, Biotechnology and Biological Sciences Research Council and Wellcome Trust, are also taking an active role in examining the necessity, justification and standards for such research (e.g. during the peer review of research grant applications) (Kolar, 2004; National Centre for the Replacement, Refinement and Reduction of Animals in Research, 2006a).

### 2.4 Special legal provisions on NHPs

In recognition of the high levels of public concern about NHP research, regulatory authorities in some countries have adopted strict measures on NHP use, effectively establishing ethical limits, giving rise to regulation which is a hybrid of **deontological** and utilitarian ethics. For example, under the UK ASPA, NHPs, together with cats, dogs and equines, can only be used where animals of no other species are suitable for achieving the scientific objective. Since 1995, there has also been an administrative ban on the use of Great Apes in scientific procedures (the UK Government will not issue licenses for their use), a ban on the use of wild-caught NHPs except where exceptionally and specifically justified, and further controls on the acquisition and use of NHPs (Home Office, 2000). Directive 86/609/EEC (European Community 1986) does not afford such special protection to NHPs (although *“degree of **neurophysiological** sensitivity”* is a consideration) but similar prohibitions are proposed for the revised Directive (see European Commission, 2008). There are special provisions under the USA Animal Welfare Act (United States Department of Agriculture, 1990) regarding environmental enhancement to promote the psychological well-being of captive NHPs, and governments in many nations have established accommodation and care requirements for these animals (Council of Europe, 2006; Home Office, 1989; National Research Council, 1996) (see Sect. 3.3).

## 3 Are NHPs worthy of special concern?

### 3.1 What are the ethical issues?

Many of the ethical issues associated with NHP use in scientific procedures are the same as those raised by the use of other vertebrate animals (Olsson et al., 2003):

- Exposure to painful or distressing scientific procedures and their effects, such as surgical interventions, infectious disease, or restraint – however, the suffering of NHPs may be different in kind from that of other animals (see Sect. 3.2);
- Housing in captive environments which limit freedom and may not meet species-typical needs, giving rise to physical or mental suffering (see Sect. 3.3) – these issues apply to breeding animals also;
- Death – very often this is required as an integral part of the experiment, because of the need to analyse tissues post mortem (see Sect. 3.4).

The purpose for which the animals are used can also raise ethical concerns (see Sect. 4.3), as can the application of new technologies (e.g. **transgenesis**: Olsson and Sandøe, 2009; Schatten and Mitalipov, 2009; Coors et al., 2010; engraftment of human neural stem cells: Greene et al., 2005).

In addition, practical issues related to the maintenance and use of NHPs raise ethical concerns that do not apply to most other animals used in research, for example:

- NHPs typically have long life spans and can spend years in captivity undergoing lengthy experiments (e.g. in behavioural neuroscience), continued use (e.g. in **pharmacokinetics**) or re-use in several independent studies during the course of their lives (see Sect. 4.2.2) (Carlsson et al., 2004; Rennie and Buchanan-Smith, 2005; Scientific Committee on Animal Health and Welfare, 2002).
- The inability of captive breeding colonies in the EU and USA to satisfy the research demand for macaques and other NHP species necessitates their importation from source countries in Asia, Africa and South America (Cohen, 2000; Hau and Schapiro, 2006; National Research Council, 2003; Prescott, 2001). The long, multi-staged journeys involved and the housing conditions, weaning and quarantine practices prior to importation can have ethical and animal welfare implications (Animal Procedures Committee, 2006; Fernstrom et al., 2008; Honess et al., 2004; Prescott and Jennings, 2004).
- Practically all of the NHPs used in research in the EU are purpose-bred, but most of the Old World monkeys used are the offspring of wild-caught parents (F1 generation) (Scientific Committee on Health and Environmental Risks, 2009). The capture and use of wild-caught NHPs for breeding for research has been criticised (Scientific Committee on Animal Health and Welfare, 2002), because of the stress, morbidity and mortality involved (International Primatological Society, 2007; Suleman et al., 1999, 2000). However, it has been argued that establishing and replenishing breeding colonies with wild-caught NHPs can be ethically justified where the animals are agricultural pests and would otherwise be killed (Stanley, 2003). To decrease reliance on wild-caught NHPs for breeding, the European Commission (2008) has proposed that after specific timelines only second generation (F2) animals born in captivity should be used in research.

### 3.2 Suffering and the moral status of NHPs

Many people, including from within the scientific community, consider that the use of NHPs in research is a matter of particular ethical concern because certain features NHPs share with humans, such as their highly developed nervous systems, cognitive complexity and intense sociality, have implications for the level or nature of suffering they might experience during experiments and are therefore morally relevant (Boyd Group, 2002; Nuffield Council on Bioethics, 2005; Weatherall et al., 2006). It is extremely difficult to determine exactly the subjective experiences of non-human animals in relation to pain and suffering. However, the evolutionary continuum that is obvious from physiological, neurological and behavioural similarities between humans, NHPs and other animals allows us to make meaningful approximations.

A great deal is known about the nervous systems of NHP species (particularly macaques) from their use in invasive neuroscience research as a model of the human brain (Peretta, 2009). It seems plausible that NHPs have the potential to experience pain in a similar way to humans, given their **neurophysiological** complexity. However, the issue is not just response to pain, but the ability to anticipate and reflect upon pain, as well as for painful memories to endure after a painful episode, which could enhance any suffering (Bateson, 1991; Bermond, 2001; Lea, 2001). The evidence for reflective self-awareness of this kind is strongest for the Great Apes (Call and Tomasello, 2008; Parker et al., 1994), which raises serious questions about the morality of using them in harmful scientific procedures (Balls, 2007; Byrne, 1999; Knight, 2008). Such considerations have probably played an instrumental role in the decisions of some countries, including the UK, the Netherlands, Austria, Belgium, Sweden, New Zealand, Australia and Japan, to ban the use of Great Apes in biomedical research or place a strong moratorium on their use. To what extent biomedical researchers from these countries make use of Great Apes in countries where they still can be used, such as the USA, is not known.

Many characteristics of the Great Apes are considered indicators of “humanhood” or “personhood”, such as their selfrecognition (and by implication self-awareness), rudimentary “**theory of mind** ”, linguistic abilities, distinct personalities, deep emotional attachments, and ability to pass on learned behaviours and customs through social mechanisms (Gómez, 1998; Scientific Committee on Animal Health and Welfare, 2002). This had led to calls to extend to Great Apes the same moral status afforded to humans and to confer on them the same legal rights as humans to life, individual liberty and freedom from torture (Anonymous, 2008; Bekoff, 1997; Cavalieri and Singer, 1993; Wise, 2000; The Great Ape Project: http://en.wikipedia.org/wiki/Great_Ape_Project).

The Old and New World monkeys more commonly used in research do not appear to share the most sophisticated mental abilities of Great Apes. Nonetheless, in the view of the Boyd Group, a forum for open exchange of views on issues of concern related to the use of animals in science “*there is strong, though not incontestable, evidence that the general richness of monkeys’ social lives and mental abilities means that compromising their way of life by using them in research and testing has the potential to cause them greater social and mental suffering than other laboratory species”* (see Boyd Group, 2002 for a discussion of the evidence).

The intense sociality of NHPs is striking and suggests that they may suffer comparatively more than other commonly-used animals from confinement and relative social isolation. Certainly, the work of Harry Harlow and others (e.g. Harlow, 1958; Law, 2009; Rosenblum and Paully, 1987) on monkey cognition and social development has demonstrated that these animals have rich subjective lives filled with intention and emotion, and that disrupting their social bonds can cause depression-like states, with obvious ethical implications (Blum, 2002; Gluck, 1997; Novak and Suomi, 1991).

The relative moral status of monkeys compared with other laboratory animals, particularly social mammals such as cats, dogs, equines and pigs, is more contentious (e.g. Webster et al., 2010). As pointed out by the Boyd Group (2002), it is difficult generally to find ways of comparing the potential for suffering of any given species with another species. Moreover, it is difficult for us, as humans, to judge capacities for suffering in, or to empathise with, species which are evolutionarily more distant from us, and it might be argued that according any species of monkey special moral status reflects human prejudice in favour of species more like ourselves.

### 3.3 Housing in captivity

NHPs are essentially non-domesticated, wild animals mostly adapted to complex tropical habitats. Confining wild animals in captivity raises ethical concerns because it imposes upon them an environment vastly different from that in which they have evolved; if they are not able to adapt to the captive conditions, this can have a serious detrimental effect on their welfare (Carlstead, 1996). There is also a view that wild animals have a right to liberty (Rachels, 1976). Although functional simulations of many aspects of the natural environments of NHPs can be replicated in captivity (Hau and Schapiro, 2004) many scientists believe there are inherent difficulties in meeting the complex social, behavioural and psychological needs of NHPs in the laboratory environment and that the minimum standards of accommodation and care established by governments in many nations may not be sufficient to provide for their physical health and psychological well-being (Boyd Group, 2002; Buchanan-Smith et al., 2004; Faucheux et al., 1978; National Research Council, 1998; Novak and Suomi, 1988; Prescott and Buchanan-Smith, 2004; Reinhardt, 2004; Savage-Rumbaugh et al., 2007; Scientific Committee on Animal Health and Welfare, 2002; Wolfensohn and Honess, 2005). Accordingly, major investments to improve housing conditions for NHPs have been made in recent years, with increased attention given to environmental enrichment and social housing, which has undoubtedly improved animal welfare (e.g. Rudling, 2003; Kelly, 2008; Waitt et al., 2008; Wolfensohn, 2008). However, there remains considerable variation in standards between establishments which has led research funding bodies to develop their own higher standards for NHP research and to make adherence to them a condition of funding, wherever the research is conducted (Laboratory Animal Science Association/Medical Research Council, 2004; National Centre for the Replacement, Refinement and Reduction of Animals in Research, 2006b).

### 3.4 Fate of the animals

The majority of NHPs used in experiments are euthanized, either because their tissues are required as part of the experiment or on compassionate grounds to alleviate unnecessary suffering. Whether it is morally wrong to prematurely end an animal’s life is a subject of philosophical debate and beyond the scope of this chapter (see Regan, 1975). Apart from the philosophical question of whether an animal is harmed by being killed, in the case of highly sociable animals such as NHPs, the implications for other members of the social group of losing a group member also may raise ethical concerns.

In situations where death is not required, for example, in the case of surplus ex-research or ex-breeding NHPs, it is often possible to “retire” the animals and allow them to live out their natural life spans (Brent, 2004; Kerwin, 2006; Prescott, 2006; Seelig and Truitt, 1999). Some establishments choose this option on ethical grounds, where it is in the best interests of the animals concerned. NHPs are intelligent animals with which it is possible to develop strong emotional bonds (Bayne, 2002; Herzog, 2002); this can make euthanasia of NHPs difficult for staff to accept (Abbot, 2008).

In the case of chimpanzees no longer needed for biomedical research in the USA, retirement is a legal requirement under the 2000 Chimpanzee Health Improvement, Maintenance, and Protection Act. This Act established a system of sanctuaries to provide lifetime care for surplus chimpanzees, none of which may be subjected to euthanasia (except where it is in the best interests of the chimpanzee involved).

## 4 Conducting ethical evaluations of NHP use

Given the high level of concern about NHP use, it is important that ethical evaluations of primate experiments are robust and thorough. This requires case-by-case scrutiny of the necessity and justification for the use of NHPs, taking into account the importance of the science, the likelihood of success, the availability of alternatives, the real “added value” of NHPs over and above other species and methods, the number of animals to be used, and the total harms caused to the animals throughout their lifetimes. Only by considering these issues together can truly informed decisions be made about whether or not certain uses of NHPs are necessary, justified and ethical. Some considerations are given below, drawn from the author’s experience. The focus is on NHP use in scientific procedures, but it is worth noting that even observational studies in the field can raise ethical issues, particularly if provisioning, capture or marking are involved (Fedigan, 2010; Gillespie et al., 2009; Jolly et al., 2003).

### 4.1 Is the NHP use scientifically necessary?

In order to establish whether NHP use is scientifically necessary, the researcher should set out in detail the reasons why he/she believes that the particular scientific objective cannot be achieved by means other than the use of NHPs, or why NHPs offer very significant scientific advantages over all other possible alternative approaches (e.g. significantly improved predictive value). The importance of achieving the objective (e.g. in terms of the clinical need or commercial interests) is not relevant in this context. The rationale for NHP use should be critically examined by independent experts with a wide knowledge of the research field in question, including all available alternative approaches – not just those based on NHPs; this may require a wider than normal pool of scientific referees.

In most fields of research where NHPs are used, the scientific justification given for their use concerns their close similarity to humans, which it is argued makes them the best available model for defined scientific questions. However, generic appeals to this similarity should not be considered sufficient justification for NHP use. Instead, the rationale should be specific and founded on robust scientific considerations, such as the presence only in the NHP species of the anatomical structures, pathways, cognitive abilities or behaviours of interest. References and information should be provided which support the rationale and which demonstrate an active search for alternatives.

Various regulatory guidelines on toxicity and safety assessments of pharmaceuticals recommend that NHPs should only be used when it is scientifically demonstrated that none of the alternative rodent and/or non-rodent species commonly used in safety testing are appropriate for the purpose of the study (International Conference on Harmonisation, 2009; Smith and Trennery, 2002). Therefore, proposals to use NHPs for safety testing should receive as much scrutiny as those proposing their use in biomedical and biological research. NHPs should not be used as a default species, on the assumption that they will be the only species representative of humans (or the species most representative of humans) or because they have been used previously.

One of the main factors driving a rise in NHP use worldwide is the increasing development of monoclonal antibodies (mAbs) as therapies for diseases such as cancer and other immune-related conditions. mAbs are highly target- and species-specific, so NHPs, typically cynomolgus monkeys (*Macaca fascicularis*), are often the only relevant animal model for preclinical safety studies. However, there are safety-relevant differences between NHP and human immune systems, even between chimpanzees and humans, which means that NHPs are not always relevant for predicting human safety (Muller and Brennan, 2009); even where they posses the intended drug target, the pharmacological activity may not be the same as in man (Chapman et al., 2009, 2010). Where NHP use is necessary, careful thought should be given to species selection, taking into account scientific, animal welfare and practical considerations (Boyd Group, 2002; Smith et al., 2001).

### 4.2 Have the 3Rs been applied fully?

Widespread support for the 3Rs principle does not always translate into action on the ground, for a variety of reasons (e.g. Coulter et al., 2009; Lloyd et al., 2008; Prescott and Buchanan-Smith, 2007). Effective implementation of all three “R”s requires researchers, regulators and members of scientific and ethical review committees to be aware of existing 3Rs approaches and methods, to put the knowledge base into practice (not just around individual experiments, but also whole research programmes and strategies), and to keep abreast of developments in science and technology that can impact on the 3Rs.

#### 4.2.1 Replacement

Opportunities for replacing the use of NHPs in research and testing are currently limited, although *in vitro* methods, human volunteers, and genetically-altered rodents all have potential (Scientific Committee on Health and Environmental Risks, 2009). A more concerted and collaborative effort is needed to accelerate the development of replacement alternatives to NHP use, since this is the only way that the associated ethical issues can be addressed wholesale; this often gets overlooked in the rhetoric surrounding NHP use.

The use of rodents or other vertebrates in place of NHPs is not replacement as defined by Russell and Burch (1959) but may be ethically desirable if an assessment of the available evidence suggests that the non-primate species is likely to suffer less harm. The judgements in such cases can be complex: for example, the transgenic mouse model for **neurovirluence** and potency testing of poliomyelitis vaccines avoids NHPs but involves greater numbers of animals and more severe endpoints (Dragunsky et al., 2003).

#### 4.2.2 Reduction

There is considerable scope for reduction where NHP use is currently unavoidable. Appropriate design of experiments is critical and greater consideration should be given to this during peer review of research proposals and scientific manuscripts (Kilkenny et al., 2009). The number of animals used in each experiment should be the minimum sufficient to answer the question posed, and researchers should justify the number of animals required, including sample size calculations where appropriate. Estimates of the number of animals needed should, where possible, take into account the required statistical significance and power level, the likely magnitude of the treatment effect (or other outcomes), the population variance and the factors that might affect this. Opportunities to further reduce the number of animals used, for example by careful planning and scheduling of breeding and experiments, should be exploited.

Sharing of study designs, data and experience, particularly in industry, can lead to significant reductions in NHP use (even without the need for regulatory change). For example, a data-sharing collaboration between the NC3Rs and pharmaceutical and biotechnology companies world-wide has identified opportunities to up to halve the number of NHPs used in the development of mAbs by decreasing the number of dose groups, recovery animals and chronic studies performed (Chapman et al., 2009). Hence, it is important to adopt a flexible, case-by-case approach to study design and drug development, based on strong scientific rationales.

Exploitation of modern technologies (e.g. *in vivo* imaging, telemetry systems, multi-unit electrophysiological recording techniques) can lead to reduction, for example, through increased data yield per animal and/or experiment (Baker et al., 1999; Kinter and Johnsen, 1999; t’Hart et al., 2006). Banking and sharing of tissues within and between establishments is another means of optimising and reducing NHP use.

The re-use of NHPs can decrease the number of animals used overall and may be driven by ethical, practical and economic considerations. However, there are ethical considerations against as well as in favour of re-use (van Vlissingen, 1999). The actual or potential harms to the animals concerned (e.g. from long-term housing and the cumulative effects of previous procedures) must be weighed against the welfare cost of obtaining and housing (and in some cases surgically-preparing) naïve animals. In the UK, re-use is subject to legal constraints (Home Office, 2000) and a reduction in the overall number of NHPs used is not considered to justify causing a significant increase in harms for individual animals. Similarly, the European Commission (2008) proposes to restrict the circumstances in which animals can be re-used in order to limit the harm caused to individual animals.

#### 4.2.3 Refinement

Refinement is misunderstood by many researchers (National Centre for the Replacement, Refinement and Reduction of Animals in Research, 2008); it refers to any approach which avoids or minimises the actual or potential pain, distress and other adverse effects experienced at any time during the life of the animals involved, and which enhances their wellbeing (Buchanan-Smith et al., 2005). Refinement is important not just for ethical reasons, but also for scientific reasons because an animal’s welfare state can affect its suitability as a research model. Developments in animal welfare science are providing increasingly more sophisticated and reliable measures of animal suffering and well-being (e.g. Mendl et al., 2009).

Many opportunities exist to refine the use and care of NHPs and much guidance is available in the scientific literature (see Rennie and Buchanan-Smith, 2006a, b, c and Joint Working Group on Refinement, 2009 for recent reviews). Researchers should ensure that every aspect of the lifetime experience of the animals is refined, including sourcing and transport; housing and husbandry; experimental design and techniques; handling; care of the animals before, during and after each procedure; end-points of the procedures; and method of killing (or other fate at the end of the experiments). The possibilities for further refinements should be continually reviewed throughout the research programme.

The high intelligence of NHPs permits behavioural management techniques to be used to reduce the amount of stress experienced during capture, transport, maintenance and research use; such techniques should be integrated into human-NHP interactions (Prescott and Buchanan-Smith, 2003; Prescott et al., 2005; Schapiro et al., 2005). Establishing appropriate relationships with NHPs is important for animal welfare generally and is of special relevance to many types of NHP research where the researchers depend on the co-operation of the animal to perform behavioural and cognitive tasks (Prescott et al., 2010)

### 4.3 Is the NHP use morally justified?

Even where it is necessary to use NHPs to achieve a particular scientific objective, and the 3Rs have been full applied, it does not mean that it is right to do so. What one person may consider a morally justified use of NHPs another may not (e.g. see the exchange of views on stroke research: Degeling and Johnson, 2009; Fox, 2009; Gerrek, 2009; Nobis, 2009; Sughrue et al., 2009a, b; and endotoxic shock: James, 2006; Wolfensohn et al., 2006; Yin et al., 2005, 2006). In practice, the test of the moral justifiability of NHP use in scientific procedures is whether or not the likely harms caused to the NHPs involved are outweighed by the anticipated benefit for humans (or other animals or the environment) A critical question is: what counts as a significantly important benefit?

A focus of the animal protection community has been the use of NHPs in fundamental research. Such research produces information that may come to be useful in understanding and treatment of disease, but is mainly pursued with the aim of advancing general knowledge in the biological sciences. For example, much neuroscience research using NHPs is conducted to understand how the structure and function of the brain contributes to perception, thinking, emotion and motor control (e.g. how brain circuits enable us to see, remember what we have seen, or to reach out and grasp an object). Some people deny that such experimentation plays a vital role in the delivery of substantial new human health benefits, or consider it to have less value than applied research (German, 2008; Martin, 2009; Sauer, 2004; Schiermeier, 2008). After lobbying on this issue by animal protection groups, the European Commission (2008) proposed to limit NHP use under the revised Directive 86/609/EEC to procedures “*undertaken with a view to the avoidance, prevention, diagnosis or treatment of life-threatening or debilitating clinical conditions in human beings* ”, but faced counterlobbying by the bioscience community (Olsson and Vitale, 2010). Whilst there have been serendipitous medical advances stemming from the unexpected outcomes of fundamental research, there has never been a robust and systematic retrospective review of the value and impact of such research and whether the scientific advances in the field have been solely dependent on the use of NHPs. This makes generic statements about NHP use being essential for improving human health difficult to substantiate.

In order for a robust harm-benefit assessment to be undertaken, the researcher should set out the anticipated benefits in precise and realistic terms, the likelihood of success, all of the harms caused to the animals (with an indication of the nature, frequency, duration and overall severity of animal suffering), any ethical issues arising from the proposed work, and why he/she personally considers that the potential benefits outweigh the harms. Members of the ethics committee must then make their own judgements about whether the likely human dividends are substantial enough to outweigh the animal suffering (for practical guidance on making such judgements, see Animal Procedures Committee, 2003; Smith and Boyd, 1991). Discussion and debate between committee members will help to clarify the issues and decide opinions. There may be disagreement about what is morally acceptable, in which case the consensus view is usually adopted. Whatever the ultimate decision, it should be defensible in the public arena.

## Glossary

### Abbreviations

Deontological ethics: An approach to ethics that judges the morality of an action based on the action’s adherence to a rule or rules. Deontologists look at rules and duties
Transgenesis: The process of introducing foreign DNA into a genome
Pharmacokinetics: A branch of pharmacology dedicated to the determination of the fate of substances administered externally to a living organism
Phylogenetic: Relating to or based on evolutionary development or history
Neurophysiological: Relating to the function of the nervous system
Neurovirulence: The tendency or capacity of a microorganism to cause disease of the nervous system
Theory of mind: The ability to attribute mental states (e.g. beliefs, desires and intentions) to oneself and others and to understand that others have beliefs, desires and intentions that are different from one’s own
Utilitarianism: The idea that the moral worth of an action is determined solely by its utility in providing happiness or pleasure as summed among all sentient beings. It is thus a form of consequentialism meaning that the moral worth of an action is determined by its outcome

## Footnotes

1 That is not to say that NHP models do not have limitations (e.g. Lewis and Johnson, 1995).

2 The last recorded Great Ape use in the EU was in 1999 (6 chimpanzees).

3 The Institutional Animal Care and Use Committees required in the USA under the amended 1966 Animal Welfare Act and 1985 Health Research Extension Act are concerned with animal care and use; not ethics.
